# Tympanostomy Tube Insertion Versus Myringotomy or Observation in Managing Otitis Media With Effusion Following Radiotherapy for Nasopharyngeal Carcinoma: An Updated Review

**DOI:** 10.7759/cureus.56905

**Published:** 2024-03-25

**Authors:** Lana M Saleh, Mohammed G Aly, Alhanouf Alhedaithy, Saleh AlAli

**Affiliations:** 1 Otolaryngology - Head and Neck Surgery, King Fahd Hospital of the University, Khobar, SAU; 2 Otolaryngology - Head and Neck Surgery, John Hopkins Aramco Hospital, Dhahran, SAU; 3 Otolaryngology - Head and Neck Surgery, King Fahad Medical Military Complex, Dhahran, SAU

**Keywords:** head and neck and radiotherapy, myringotomy, tympanostomy tube, otitis media with effusion, post-radiotherapy, nasopharyngeal cancer (npc)

## Abstract

Head and neck cancers, including nasopharyngeal carcinoma (NPC), are relatively common in Saudi Arabia. Radiotherapy is a standard treatment for NPC, but it can lead to side effects, including post-radiation otitis media with effusion (OME). Managing post-radiotherapy OME remains a topic of debate, with various interventions proposed. This study aims to review the efficacy of different methods to manage post-radiotherapy OME in NPC. This includes tympanostomy tube insertion, frequent myringotomies, and observation. A systematic review was carried out for articles published between 1975 and 2023 following the Preferred Reporting Items for Systematic Reviews and Meta-Analyses (PRISMA) guidelines. Excluded from the analysis were articles that involved patients undergoing surgical treatment for nasopharyngeal cancer, studies that focused on patients with other head and neck cancers who developed OME after radiotherapy, research investigating the effectiveness of surgical procedures unrelated to tympanostomy tube insertion, studies written in non-English language, and case reports, reviews, or conference letters. A total of 450 studies were screened, of which six studies were included in the review, yielding 328 patients. The mean age ranged between 46 and 52 years. Follow-up varied from six months to 11 years. The intervention in all studies was tympanostomy tube insertion, and the controls were myringotomy, observation, or tympanic membrane fenestration with cauterization. The use of recurrent myringotomies for the treatment of OME in patients with NP post-radiotherapy is associated with improved chances for the resolution of effusion and decreased risk of complications when compared to tympanostomy tube insertion. Hence, we recommend following a step-wise approach when dealing with this group of patients, offering grommets for patients with persistent effusion or those who cannot tolerate frequent procedures.

## Introduction and background

Head and neck cancers constitute 6% of all malignancies in Saudi Arabia, and one-third of these cases are nasopharyngeal carcinoma (NPC) [1]. The primary treatment for nasopharyngeal cancer typically involves radiotherapy, but this approach can lead to a range of side effects. Among the various challenges faced by these patients, post-radiation OME is a frequently encountered issue.

OME is characterized by the accumulation of fluid in the middle ear without an acute infection [2]. In the context of post-radiation, OME occurs due to swelling and impaired function of the eustachian tube (ET). The management of OME in patients who have undergone radiation therapy for nasopharyngeal cancer has been a subject of controversy, with no clear consensus on the most effective treatment approach [3].

Several interventions have been proposed to alleviate OME symptoms in patients with NPC post-radiotherapy. These interventions include watchful waiting, regular myringotomies using a blade or laser, myringotomy with tympanostomy tube insertion, and balloon dilatation of the ET. Each of these approaches offers certain benefits but also comes with an increased risk of specific complications, adding to the overall controversy surrounding this topic [3-7].

Although tympanostomy tube insertion for OME after radiation therapy provides immediate relief of symptoms, this intervention has been associated with a higher incidence of chronic otitis media (COM) [3-5].

The most recent systematic review addressing this issue was conducted in 2016 by Schwarz et al. [3]. This review focused on the effectiveness of tympanostomy tube insertion in treating OME following radiotherapy for NPC and recommended a gradual approach to managing these patients. This approach begins with observation and keeping surgical interventions for patients with more persistent and troublesome symptoms [3].

The purpose of this review is to evaluate the efficacy of different modalities of treating post-radiotherapy OME and the associated complications in patients with NPC, which are considered prevalent malignancies in the local community.

Methods

This review followed the PRISMA guidelines.

Search Strategy

The bibliographic search was carried out in the following databases for articles published between 1975 and 2023: PubMed, Cochrane, and ProQuest. PubMed database was searched using specific descriptors and their synonyms according to Medical Subject Headings: NPC, nasopharyngeal, nasopharyngeal neoplasms, nasopharyngeal cancer, radiotherapy, radiation therapy, intensity-modulated radiotherapy, conformal radiotherapy, otitis, otitis media, and otitis media with effusion. The terms were associated with one another with the words AND and OR. A similar search strategy was carried out in Cochrane and ProQuest. The reference lists cited in systematic reviews and eligible studies were examined. The searches were conducted between September 18 and September 25, 2023.

Selection Criteria

All retrieved studies were screened for title, abstract, and/or full text to make sure that they meet the objective of the review. Two independent researchers critically assessed the articles for eligibility. Exclusion criteria included studies written in languages other than English, case reports, reviews, or conference letters; no abstract or full text available; studies including patients who received surgical management for nasopharyngeal cancer; articles including other head and neck cancer patients with OME post-radiotherapy; and studies assessing the efficacy of surgical interventions other than tympanostomy tube insertion. The eligible studies were thoroughly reviewed by two independent reviewers to collect the required data.

Data Extraction and Outcome Measures

The basic characteristics of each study population were extracted, including the number of patients or ears included, the mean age of the study population, and the mean follow-up period. Then, the primary outcome of interest was the resolution of OME. Secondary outcomes were based on the incidence of complications, mainly recurrent or persistent OME, and the incidence of COM. The number and percentage representing the outcome of interest were pooled from the included studies.

Risk-of-Bias Assessment

The quality of the selected studies was evaluated using the revised Cochrane risk-of-bias tool [6] for the randomized clinical trial (RCT). The following domains were evaluated and scored as low, unclear, or high risk of bias: randomization process, deviation from intended interventions, missing outcome data, measurement of the outcome, selection of the reported results, and overall bias. The risk of bias in cohort studies was evaluated using the Newcastle-Ottawa scale [7]. The following domains were assessed: selection, comparability, and outcomes. Each domain has multiple items, and the study is considered high-risk for bias if it failed with one or more items.

Statistical Analysis

Pooled odds ratios (ORs) and 95% confidence intervals (CIs) were calculated using random-effects models due to anticipated heterogeneity. Heterogeneity was assessed using I^2^ to quantify inconsistency and Q tests. Funnel plots assessed publication bias, with Egger’s test not applicable due to the small sample. Analysis was conducted in RStudio version 2023.06.0+421 using the meta package (RStudio Team (2020), RStudio: Integrated Development for R. RStudio, PBC, Boston, MA, URL: http://www.rstudio.com/).

Results

Search Results

**Figure 1:**
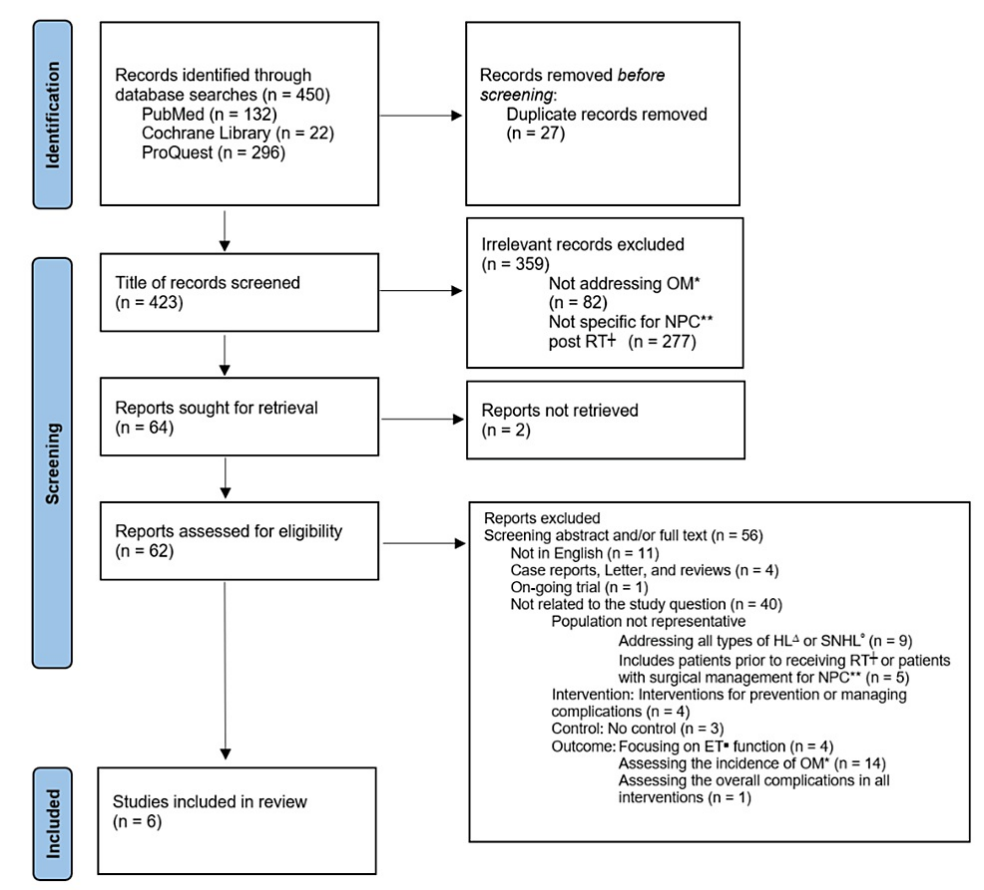
PRISMA 2020 flow diagram * OM: otitis media, ** NPC: nasopharyngeal cancer, ┼ RT: radiotherapy, ∆ HL: hearing loss, ⁰ SNHL: sensorineural hearing loss, ■ ET: Eustachian tube, PRISMA: Preferred Reporting Items for Systematic Reviews and Meta-Analyses

The literature search resulted in a list of 423 records after the removal of duplicates. A total of 359 were excluded after screening the title. Two studies were excluded as the full-text articles could not be retrieved. An additional 16 articles were excluded after abstract or full-text screening. Forty were excluded after more critical appraisal following PICO (population, intervention, control, and outcomes) guidelines from evidence-based medicine, leading to six included studies (Figure 1).

Characteristics of the Included Studies

Of the included studies, two were RCTs and four were cohort studies (two were retrospective and the other two were prospective). The number of patients per study ranged from 18 to 96. The mean age varied between the studies from 46 years to 52 years. The follow-up time differed from six months to 11 years. The intervention in all studies was tympanostomy tube insertion, and the control group encompassed different forms of management, including myringotomy, observation, and other tympanic membrane procedures. The characteristics of each study are demonstrated in Table 1.

**Table 1:** Characteristics of studies included in the systemic review between 1975 and 2023 * Tympanic membrane fenestration with cauterization, NA: not applicable

Authors (year)	Country	Study design	Mean age (years)	Mean follow-up	No. of patients/ears	Intervention (no. of patients/ears)	Control (no. of patients/ears)		
							A	B	C
Chen et al. [4](2001)	Taiwan	Retrospective cohort	46	11 years	67/100	Grommet insertion (27/40)	Myringotomies (40/60)	NA	NA
Charusripan et al. [5](2017)	Thailand	Prospective randomized controlled trial	49.6	6 months	43/43	Grommet insertion (20/20)	NA	Observation (23/23)	NA
Liang et al. [8](2010)	Taiwan	Prospective cohort	46.1	842.1 days	85/124	Grommet insertion (23/46)	Myringotomy (55/110)	Observation +/- HA (7/14)	NA
Xu et al. [9](2008)	China	Prospective quasirandomized clinical trial	48.8	2 years	96/135	Grommet insertion (NA/45)	Myringotomy (NA/45)	NA	Others^*^ (NA/45)
Young et al. [10] (1998)	Taiwan	Prospective cohort	51	5 years	19/20	Grommet insertion (NA/9)	Myringotomy (NA/11)	NA	NA
Young et al.[11] (1995)	Taiwan	Retrospective cohort	52	5 years	18/18	Grommet insertion (NA/9)	Myringotomy (NA/9)	NA	NA

Risk of Bias in Studies

The quality assessment of the included studies is shown in Table 2 and Table 3. Overall, the risk of bias for the cohort studies was mild. One RCT showed an uncertain risk of bias due to the inadequate reporting of the allocation process and the method of analysis.

**Table 2:** Quality assessment of the included cohort studies based on the Newcastle-Ottawa scale Each asterisk represents 1 point in each field of the Newcastle-Ottawa Quality Assessment Scale as follows: * one point, ** two points, *** three points, **** four points.

Author	Selection	Comparability	Outcome
Chen et al. [4]	***	**	***
Liang et al. [8]	****		***
Young et al. [10]	****		***
Young et al. [11]	****		***

**Table 3:** Quality assessment of the included randomized clinical trial studies based on the Cochrane risk-of-bias tool Risk of bias based on the Cochrane risk-of-bias tool as follows: + low risk, ? some concern.

Author	Randomization process	Deviation from intended interventions		Missing outcome data	Measurement of the outcome	Selection of the reported results	Overall bias
		Effect of assignment to the intervention	Effect of adhering to the intervention				
Charusripan et al. [5]	+	+	+	+	+	+	+
Xu et al. [9]	?	?	+	+	+	+	?

Meta-Analysis of the Results

This meta-analysis included six studies investigating the relationship between tympanostomy tube insertion and other control groups (myringotomies, observation, and tympanic membrane fenestration with cauterization) on resolution/persistence and recurrence of OME and incidence of COM. The random effects model was used to estimate the overall effect size.

Resolution of OME

**Figure 2:**
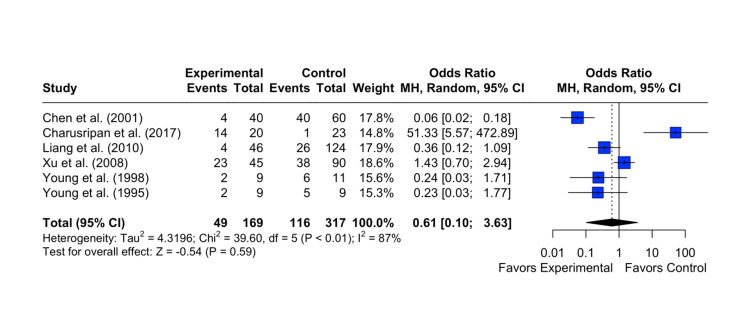
Effect of grommet insertion on the resolution of otitis media with effusion in patients with nasopharyngeal carcinoma post radiotherapy compared to observation, myringotomies, and tympanic membrane fenestration Citations: Chen et al. (2001) [4], Charusripan et al. (2017) [5], Liang et al. (2010) [8], Xu et al. (2008) [9], Young et al. (1998) [10], and Young et al.(1995) [11]

The results showed that the pooled OR was 0.61 with a 95% CI of [0.10; 3.63]. The z-test for the overall effect size was not statistically significant (p = 0.59), indicating that there was no significant difference between tympanostomy insertion and the control group on the resolution of OME. The analysis also revealed high heterogeneity among the studies, with an I^2^ statistic of 87%, indicating substantial variability between the study results. The test of heterogeneity was statistically significant (p < 0.0001) (Figure 2).

Persistence of OME

**Figure 3:**
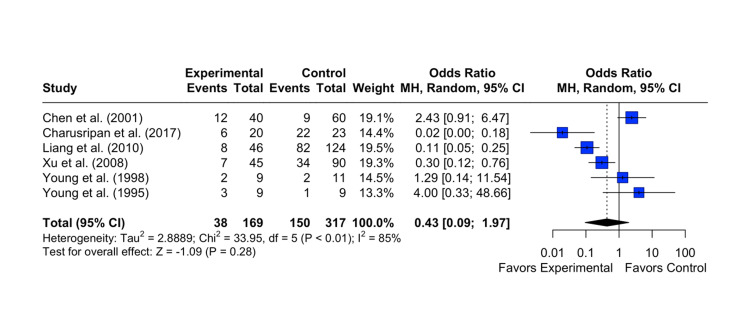
Effect of grommet insertion on the persistence/recurrence of otitis media with effusion in patients with nasopharyngeal carcinoma post radiotherapy compared to observation, myringotomies, and tympanic membrane fenestration Citations: Chen et al. (2001) [4], Charusripan et al. (2017) [5], Liang et al. (2010) [8], Xu et al. (2008) [9], Young et al. (1998) [10], and Young et al.(1995) [11]

The results showed a pooled OR of 0.43 with a 95% CI of [0.09; 1.97]. The z-test for the overall effect size was not statistically significant (p = 0.28), indicating no significant association between the intervention and the control group on the persistence of OME. Heterogeneity among the studies was observed, with an I^2^ statistic of 86%, suggesting substantial variability in the study results. The test of heterogeneity was statistically significant (p < 0.0001) (Figure 3).

COM

**Figure 4:**
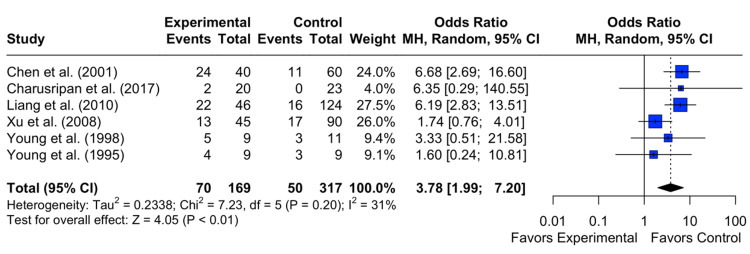
Effect of grommet insertion for otitis media with effusion in patients with nasopharyngeal carcinoma post radiotherapy on incidence of chronic otitis media compared to observation, myringotomies, and tympanic membrane fenestration Citations: Chen et al.(2001) [4], Charusripan et al.(2017) [5], Liang et al.(2010) [8], Xu et al.(2008) [9], Young et al.(1998) [10] and Young et al.(1995) [11]

The results revealed a pooled OR of 3.78 with a 95% CI of [1.99; 7.20]. The z-test for the overall effect size was highly statistically significant (p < 0.0001), indicating a significant difference between the intervention and the control group on the incidence of COM. Heterogeneity among the studies was minimal, with an I^2^ statistic of 31%, suggesting moderate variability in the study results. The test of heterogeneity was not statistically significant (p = 0.20) (Figure 4).

Subgroup Meta-Analysis

**Figure 5:**
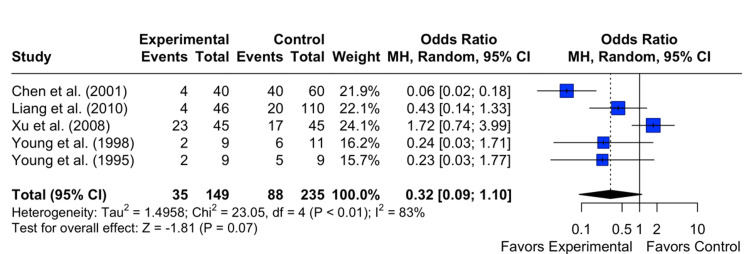
The odds of resolution of otitis media with effusion in patients with nasopharyngeal cancer post radiotherapy in those who received grommet insertion compared to myringotomies Citations: Chen et al. (2001) [4], Liang et al. (2010) [8], Xu et al. (2008) [9], Young et al. (1998) [10], and Young et al. (1995) [11]

**Figure 6:**
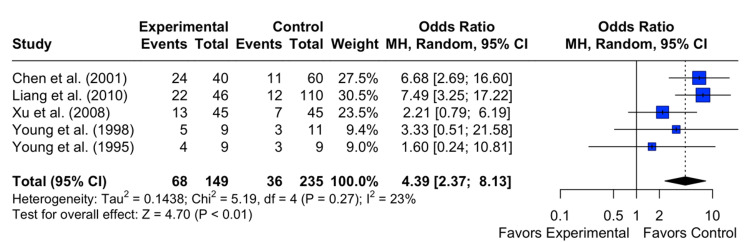
Odds of COM in patients who received grommet insertion for otitis media with effusion post radiotherapy for nasopharyngeal cancer compared to those who received myringotomies Citations: Chen et al. (2001) [4], Liang et al. (2010) [8], Xu et al. (2008) [9], Young et al. (1998) [10], and Young et al. (1995) [11]

Subgroup meta-analyses were conducted to compare the intervention (tympanostomy tube insertion) with myringotomies. The analyses revealed a non-significant difference between the two groups on the resolution of OME (OR 0.3157, 95% CI 0.0907 to 1.0992, I^2^ = 82.6%, p < 0.0001) (Figure 5) and on the incidence of persistent/recurrent OME (OR 0.6320, 95% CI 0.1442 to 2.7705, I^2 ^= 86.9%, p < 0.0001). However, a significant increased risk of incidence of COM was demonstrated when comparing tympanostomy tube insertion versus myringotomies (OR 4.3878, 95% CI 2.3684 to 8.1289, I^2 ^= 22.9%, p = 0.2683) (Figure 6). In summary, the subgroup analysis revealed that when comparing the myringotomy control group with the intervention, there is no difference between the two groups in the resolution and persistence/recurrence of OME. However, there is a significant difference in the development of COM.

Publication Bias

**Figure 7:**
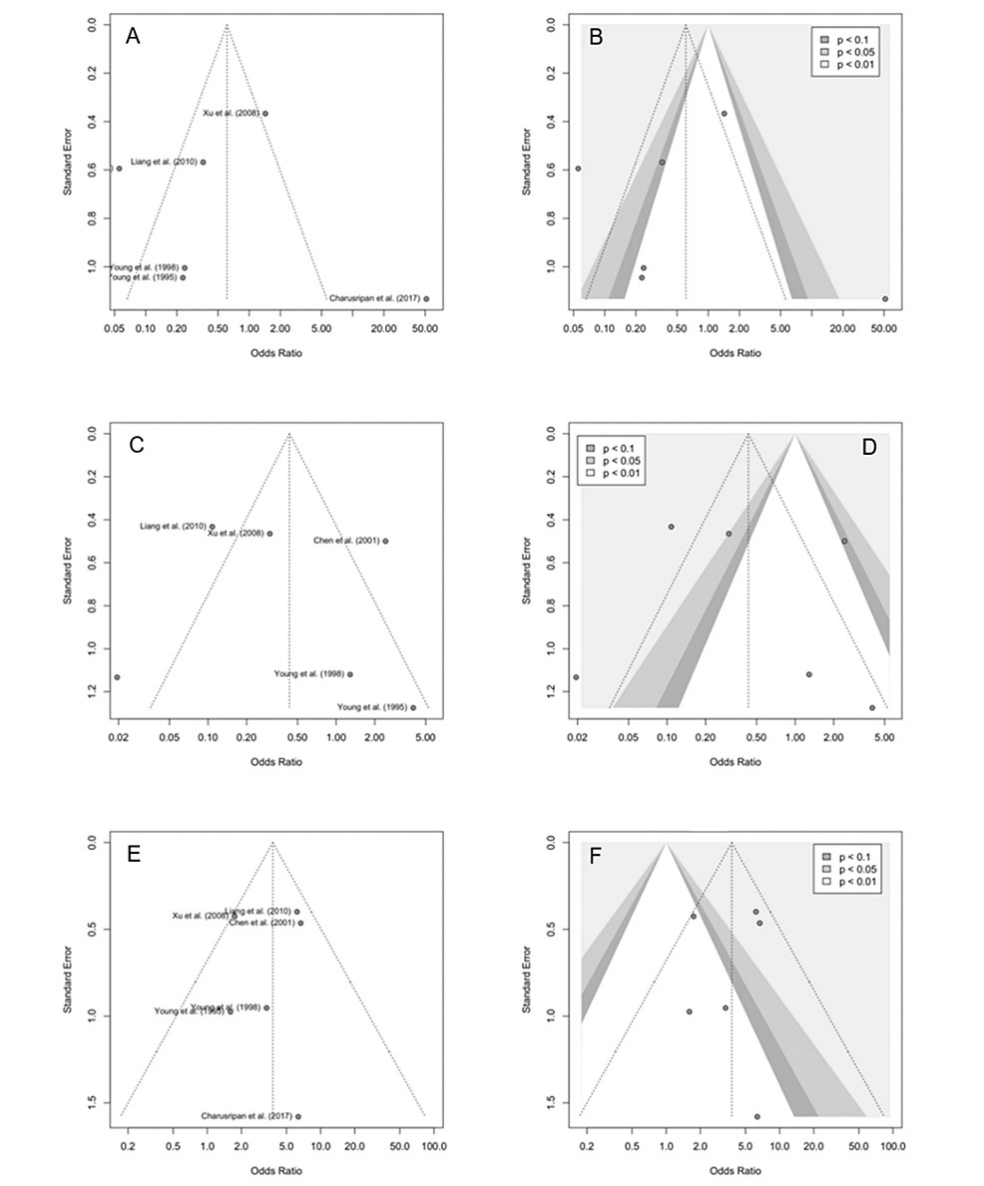
Funnel plots and color-enhanced funnel plots for the publication bias assessment of meta-analyses Graph A: funnel plot (resolution of OME), Graph B: contour-enhanced funnel plot (resolution of OME), Graph C: funnel plot (persistent recurrent OME), Graph D: contour-enhanced funnel plot (persistent recurrent OME), Graph E: funnel plot (COSM), Graph F: contour-enhanced funnel plot (COSM) Citations: Chen et al. (2001) [4], Charusripan et al. (2017) [5], Liang et al. (2010) [8], Xu et al. (2008) [9], Young et al. (1998) [10], and Young et al.(1995) [11]

Funnel plots assessed the publication bias for the three outcomes. For the resolution of OME, the plot showed asymmetry with two small studies at the bottom left and two outliers, while enhancement identified two studies at p < 0.01, suggesting small study effects. The persistent OME plot also demonstrated asymmetry, with two studies at the bottom right and three outliers. Enhancement found one at p < 0.05 and two at p < 0.01, supporting potential small study effects. The COM funnel plot appeared more symmetrical with a spread of studies, although one small study lay at the bottom and two larger ones at the middle. Enhancement identified two studies at p < 0.01 (Figure 7).

## Review

Post-radiotherapy OME symptoms, such as earache, tinnitus, and reduced hearing, may affect the quality of life significantly. The incidence of OME within five years following the completion of radiotherapy in individuals with nasopharyngeal cancer has been reported to reach 29% [12]. Various pathophysiological mechanisms have been proposed to account for OME in this patient population, encompassing both organic and functional origins. Nevertheless, the prevailing consensus attributes its cause to inflammation and fibrosis in the nasopharynx, resulting in ET dysfunction and compromised gas exchange in the middle ear [13-14].

Young et al. have documented that ET dysfunction following radiation is not permanent, with functional impairment showing a recovery rate of 50% and 90% at the five- and 10-year marks, respectively [14]. Nonetheless, due to the considerable impact of OME on patients' well-being, many individuals seek medical intervention to alleviate their distress, leading to the development of various treatment strategies for this condition.

The resolution of OME was found to be variable when comparing tympanostomy tube insertion to the different control groups. However, the result of the subgroup meta-analysis revealed that patients who underwent repeated myringotomies had a higher likelihood of OME resolution and a reduced risk of recurrent or persistent effusion compared to those who underwent tympanostomy tube insertion. Similar results were observed in studies comparing these two groups of management, with outcomes favoring frequent myringotomies over tympanostomy tubes [4,10-11]. It has been suggested by Young et al. that the persistence of effusion in the intervention group might be attributed to superinfection, as tympanostomy tube insertion can forcibly open the ET, potentially allowing infection to spread from the nasopharynx to the middle ear [14].

The risk of developing COM was notably elevated in the intervention group when compared to overall control groups in general and to frequent myringotomies specifically. As a result, many studies recommend retaining the use of grommets in cases with severe and persistent symptoms [4,9-11]. Recently, the utilization of balloon dilatation of the ET has been suggested as an alternative to tympanostomy tubes for patients post-radiation therapy. Nevertheless, the effectiveness of this intervention was observed to be limited and provided relief for only a short duration [15].

One limitation of this systematic review and meta-analysis is that all the studies included in the review focused on the Eastern Asian population. This could be attributed to the higher prevalence of nasopharyngeal cancer in this region. However, it is worth noting that some articles were initially excluded due to being written in non-English languages, with most of these articles being in Chinese. The exclusion of these non-English language articles may have an impact on the overall results and should be considered when interpreting the findings.

## Conclusions

In summary, this metanalysis systematic review supports the use of myringotomies and aspiration of middle ear effusion as a first step for the management of postradiotherapy OME in nasopharyngeal carcinoma patients rather than insertion of tympanostomy tubes. Both approaches have similar outcomes with the possibility of enhanced outcomes in the former group, in addition to the low rate of otologic complications associated with the myringotomies with tympanostomy tube insertion. However, it is essential to approach each case individually, as some patients, such as the elderly or those with low pain tolerance, may be better to performing tympanostomy tube insertion to minimize the need for more frequent interventions. Additional research is warranted to determine the optimal number of myringotomies required in these patients until ET function is restored and to assess the effectiveness of interventions involving the ET in comparison to myringotomies and tympanostomy tube insertion.
